# Designing Inclusive Qualitative Research with Carers of People Living with Dementia: Methodological Insights

**DOI:** 10.3390/healthcare11152125

**Published:** 2023-07-25

**Authors:** Jessica Runacres, Daniel Herron

**Affiliations:** 1 Department of Midwifery and Allied Health, Staffordshire University, Staffordshire ST4 2DE, UK; 2 Department of Psychology, Staffordshire University, Staffordshire ST4 2DE, UK; daniel.herron1@staffs.ac.uk

**Keywords:** informal carers, caregivers, dementia, qualitative research, inclusive, nominal groups technique, focus group

## Abstract

The support provided by carers of people living with dementia results in savings for the UK economy; however, providing this care has a significant impact on carers. Supports are needed to ensure that carers can continue to provide care, and carers should be involved in the generation of the evidence necessary to develop such support. However, this relies on their ability to meaningfully engage with research, yet current data collection methods create obstacles to engagement. In this paper, we aim to provide a critical examination of approaches to qualitative data collection with carers and produce recommendations for the design of inclusive research. First, different approaches to qualitative data collection are discussed and appraised. Following this, a case study of inclusive research is presented, illustrating how carers can be facilitated to engage in research. Finally, recommendations for inclusive research are offered, including the collection of data without the cared-for person present, building additional care into a study design, providing ‘incidental funds,’ offering sustenance and remuneration, and undertaking research in a neutral space. These recommendations are designed to facilitate the involvement of carers in research and promote the use of more varied or multifaceted methods to develop the current evidence base.

## 1. Introduction

As the population ages and people live longer, dementia and the provision of care for those living with dementia have become a growing challenge. In the UK, it is estimated that roughly 850,000 people are living with dementia [1], and, in developed countries, it is estimated that those living with dementia will nearly double to 14.3 million by 2050 [2]. Dementia is one of the most expensive conditions in the world, particularly towards the end of life [3]. Care for people living with dementia costs the UK economy £34.7 billion, and this is projected to rise to £94.1 billion by 2040 [4]. These figures include costs to the NHS, social care costs (e.g., residential and home care), and the cost of unpaid care; the largest proportion of these costs is for social care. To provide care for the rising number of individuals living with dementia, the UK government has stated that an increase in the number of people being cared for informally in their homes is needed [5]. Those who provide this care are termed ‘carers.’ The term ‘carer’ is used in this article to denote those who provide unpaid support for a friend or family member living with dementia; this may be as co-habitants, or the person living with dementia (PLWD) may live in their private residence or a place offering residential care. 

The support provided by carers results in vast savings for the UK economy [6]. However, providing care has a significant impact on a carer’s quality of life [7]. While some carers report that providing care strengthens their relationship with the PLWD and provides them with a sense of achievement [7,8], research has found that providing care creates a number of challenges. For example, increased financial concerns [9], stress, depression, anxiety [10], and stigma, which increase caregiver burden [11], Lindeza et al. [7] identified through a systematic review that providing care for a PLWD had a significant impact on a carer’s ability to manage their social and professional responsibilities, led to increased feelings of sadness and stress, and reduced financial wellbeing. Providing long-term care for a PLWD can also have a significant impact on a carer’s own physical health. As the PLWD experiences a worsening of symptoms and reduced physical mobility, an increase in physical support may be required from the carer [12]. To address the challenges experienced by carers, support is needed to ensure they can continue providing care. The level and quality of support received by carers have been found to be a mediating factor in their experiences [13], and it has been found that a carer who is well supported is able to provide better care and experience better outcomes for themselves [14]. 

Carers need to be involved in the development of supports as experts-by-experience to generate the evidence necessary for the implementation of effective interventions and ensure that these are appropriate for their needs [15,16]. They also have a unique insight into the needs of people living with dementia and can therefore facilitate the development of supports for this group. Consequently, carer insights are important for research. However, the success of such research often relies on the recruitment of a sufficient sample of carers and their ability to meaningfully engage, something that is challenging [17,18,19]. Leach et al. [20] utilised 16 different recruitment strategies for carers of PLWD in a study that aimed to examine the challenges of recruiting carers into community-based clinical research and investigate the needs of carers as study participants. They found that despite the broad recruitment strategies adopted (e.g., posters, social media, and radio), the study underrecruited by 53%. This is in line with other community-based trials, which have been identified as those least likely to recruit their target number of participants [21]. Leach et al. [20] explained that a major obstacle to engagement in the clinical trial was not the method of recruitment but rather the support offered around the method of data collection. 

Despite the importance of research with carers, their ability to engage is often problematic. Carers are generally time-poor, with competing demands of carer duties, personal lives, household tasks, and, for some, employment [7,22]. Much research, especially clinical trials or interventions, requires several data collection points, which may therefore prohibit some carers from participating [23,24]. Many carers of PLWD are elderly, and while they will be less likely to have employment responsibilities, they may experience additional challenges to engagement, such as mobility or access to transport [25]. 

Perhaps one of the most significant obstacles faced by carers when considering their engagement in research is respite care [24,26]. Many carers are unable to leave the PLWD on their own for an extended period [27], and they may have limited access to respite support from professional services or friends and family. Designing research that requires the carer to spend time away from the PLWD and not providing financial remuneration to cover the costs of additional care needs or providing care as part of the research design excludes carers from being able to engage in research. This is further exacerbated if there is a financial burden of the research (e.g., cost of transport, respite care, or meals), and this disproportionately impacts low-income and minority carers [22]. 

Considering the importance of carrying out research with carers of PLWD, which draws on a range of experiences from individuals with differing backgrounds, researchers need to carefully consider inclusive practices. Therefore, in this article, we aim to provide a critical examination of approaches to qualitative data collection with carers and produce recommendations for the design of inclusive research. Qualitative methods of data collection are focused on, as these enable the collection of perceptions and insights and enable the researcher to develop an in-depth understanding of a carer’s experiences. Also, qualitative methods of data collection are typically more time-consuming and therefore likely to be more exclusionary for carers. In this paper, different approaches to qualitative data collection are discussed and appraised in consideration of their use with carers of PLWD. Following this, an approach to inclusive research we undertook [28] is presented as an example of how carers of a PLWD can be facilitated to engage in qualitative research. Finally, recommendations for future inclusive research are offered.

## 2. Qualitative Data Collection Approaches with Carers of People Living with Dementia

There are multiple approaches to qualitative data collection, but to gather in-depth qualitative data with carers, focus groups, dyad interviews, and one-on-one interviews tend to be popular. We, therefore, describe and examine each of these approaches in turn in the subsequent sections, using examples from previous research with carers of PLWD to illustrate our points. Table 1 provides a summary of the strengths and limitations of each approach in relation to data collection with carers of people living with dementia.

### 2.1. Focus Groups

Focus groups are a common method of gathering data with carers [29,30,31]. They collect information from multiple participants at once and promote interaction and spontaneity by encouraging participants to share and explain their views and disagree with others. Consequently, opinions and experiences are shared that may not surface during individual interviews [32]. However, with sensitive topics, such as care duties, issues of confidentiality and anonymity are acute [33]. While the public nature of a focus group in which participants are unknown to each other may create a sense of perceived anonymity, it requires participants to share information that they might typically regard as private, and it is not possible to predict the reaction or discretion of the group [34]. Nevertheless, it is common for focus group participants not to know each other [30,31]. Bruinsma et al. [29] included multiple carers from the same family within each of their focus groups with carers. This may have made some participants feel more comfortable; however, they reported that this could have resulted in some carers feeling hesitant to discuss certain sensitive topics, and some may have provided socially desirable answers; this therefore could have limited the insights they were able to gather. 

Some focus groups with carers are comprised of only carers, and therefore participants are among peers [29,30,31]. Bruinsma et al. [29] concluded that those carers who have less access to support may have a greater need for peer support and might have been more likely to agree to participate in their focus groups; therefore, these carers may have been overrepresented in their research and impacted the data collected. However, no remuneration was offered to participants; this is common in research with carers [30,31,35]. Considering the challenges carers face in arranging support and respite care, research that does not offer financial remuneration to cover the costs of additional care or provide care as part of the research design is likely to exclude those who have less access to support, which contrasts with the conclusions of Bruinsma et al. [29]. Hudson et al. [30] support this view; they did not provide any remuneration, and they identified that there was a high level of heterogeneity in their focus groups, with most participants being white British. Hudson et al. [30] suggest that future research should include a more diverse sample of carers; this is especially important as there are influences of ethnicity and culture on carers’ experiences [36,37]. Furthermore, those carers with a higher care burden are likely to be excluded from participation due to being time poor. In research for which a carer is required to arrange alternative care for participation, an incentive should be offered to aid with travel and respite care costs. It is common for research to offer vouchers to recognise participants’ contributions [38,39], but this is inadequate for meeting costs for low-income participants.

Much research is limited in terms of the recitation and practical supports it can offer participants, and therefore, this can limit carer participation [24]. Some research utilises focus groups in which the carer and PLWD are both present [39]; this removes the need for the carer to source additional care for the PLWD. However, some carers may feel uncomfortable disclosing information about their experiences in front of the person they provide care for and may focus on the PLWD’s experiences instead of their own, as it is typical in their lives for the focus to be on the PLWD [38]. To avoid this issue while still including carers and those living with dementia in one group, Wammes et al. [35] separated carers from those they provide care for into different focus groups. While this did not remove the need for the carer to source additional care for the PLWD as groups were not concurrent, they argued that it would support free discussion. Dementia can result in significant language and communication impairments, which can be particularly acute in groups [40]. Sutcliffe et al. [39] reported that some of those living with dementia were ‘silent’ members of focus groups as impairments hindered their involvement and ability to contribute. This effect was not reported in Wammes et al. [35], but as those living with dementia were the minority in groups and their carer was not present to support them, it is likely that their experiences were not equally captured. Consequently, the conduct of focus groups in which the carer and PLWD are both present raises challenges in the collection of both the carer and PLWD’s experiences.

### 2.2. Dyad Interviews

To address some of the issues with focus groups and to collect the experiences of both the PLWD and the carer at the same time, dyad interviews can be considered. The use of dyad interviews, in which a PLWD and their carer are interviewed together, is common in dementia research [19,41,42]. The dyadic interview approach enables the care relationship, which is central to shaping experiences of dementia, to be explored [43]. The relational component of this approach to data collection is relevant where the relationship between the carer and PLWD is the focus of the research, and this is especially typical in research focusing on spousal carers [44,45,46]. However, in some studies, this approach is instead used to gather both the views of the carer and PLWD at the same time or to provide insight into areas not known by the researcher [19,41,42,47]. Dyad interviews are also commonly adopted to facilitate the participation of the PLWD by ensuring their safety and wellbeing and to support scientific integrity by acting as an informant and providing information (e.g., medical history) as necessary [48,49].

Despite the advantages of this approach, data gathered from dyad interviews may not portray an accurate representation of participants’ experiences. Research has found that more in-depth, personal, and sensitive information is shared in individual interviews compared to dyadic interviews [50]. Discussing sensitive issues can cause discomfort in a group interview setting [51], and this is likely exacerbated in dyad interviews in which both the carer and PLWD are present, as a carer may feel uncomfortable discussing their experiences of providing care in front of the individual they provide care for. Another possible drawback of dyad interviews is the potential for domination, where one participant talks more than the other, does not build on or respond to what the other has said, or is dismissive [50,51]. This issue can be particularly apparent when the power is not equally distributed between participants, much like within a caring relationship [47,52]. Finally, the use of dyad interviews when the focus is on the PLWD further increases the demand for the carer’s time [19].

### 2.3. One-on-One Interviews

To capture the experiences of carers without the presence of the PLWD, one-on-one interviews can be used. Interviews enable an in-depth exploration of participants’ experiences and allow participants to talk about issues important to them. Many interviews with carers are semi-structured [38,53,54]. These apply an interview guide in a flexible manner, allowing the interviewer to follow up on concepts raised by the participant; therefore, the interview is personalised, and the participant can frame their interview within the context of their own lives [55]. 

Face-to-face interviews are considered the ‘gold standard’ approach; in these, there are no delays due to technology, and body language, non-verbal cues, and facial expressions are obvious to the interviewer [56]. However, face-to-face interviews are costly and time consuming, and they also do not address issues raised with other methods relating to additional care needs for the PLWD and the exclusion of certain carers. Some research suggests that data collection could take place in carers’ homes to facilitate engagement [20] and to ensure the participant feels comfortable in their surroundings [57], though this raises ethical and safety concerns for elderly individuals (e.g., allowing an unknown person into a household with a vulnerable person) and the researcher (e.g., lone working) [57,58]. Virtual video interviews are thought to be similar to face-to-face interviews due to the ability to see the participant [59]. Although, unlike face-to-face interviews, there is no need to consider factors such as travel and safety, some individuals may be excluded due to limited computer literacy or access to technology [56]. This is particularly acute for spousal carers of individuals with dementia who are likely to be elderly. Undertaking research with a carer in their own home, whether this is in person or virtually, may limit the information they are willing to provide, or they may be distracted by the needs of the PLWD. For example, carers may feel uncomfortable sharing their true experiences if the person they provide care for overhears. Therefore, interviews should be conducted in a neutral space; however, this again raises the need for additional care for the PLWD to facilitate the involvement of the carer. 

### 2.4. Summary of Discussed Methods of Data Collection

As discussed, multiple strategies are adopted to gather in-depth qualitative data with carers of PLWD, yet there are issues with the implementation of these that can exclude certain demographics, therefore limiting the scope of data collected; for example, the exclusion of those from low-income households or those with a higher care burden. Of particular significance is the need to provide care for the PLWD or remuneration to cover the costs of this additional care so that the carer can express themselves without the potential for the PLWD to overhear and reduce the burden of participation on the carer. While one-on-one interviews support a personalised approach to data collection and are easier to coordinate than methods with multiple participants [55], focus groups provide interactional opportunities that can not only increase the depth and breadth of topics covered but also provide opportunities for peer support [29]. 

The design of research to ease the burden on carers and reduce the need for remuneration precludes the use of certain methods that may take longer to carry out, such as creative methods of data collection (e.g., arts-based methods) or more structured techniques (e.g., the nominal group technique). The methods adopted with carers of people living with dementia should not be limited due to practical concerns, as this reduces the scope and quality of the evidence base and is an injustice for dementia carers as their voices are not adequately represented. Instead, researchers should strive to design supportive research projects in which carers involvement is facilitated.

## 3. Inclusive Research with Carers of People Living with Dementia: A Case Study

The NIHR [60] recently published an infographic to support researchers in involving unpaid carers; this highlighted some general considerations such as being flexible, putting carers first, and reducing the burden on them; however, these are not specific to carers of a PLWD, and examples of how these concepts have been applied in practise are yet to be seen. While the NIHR [60] guidance was not published prior to its undertaking, we applied similar concepts within our research, which aimed to explore the support priorities of older (65+ years old) carers of people living with dementia [28], and this can be used as an example of inclusive qualitative research with carers. 

The data collection was undertaken by two experienced qualitative researchers. To address the aim, we designed a multi-method qualitative study in which participants first undertook an in-person focus group with other dementia carers previously unknown to them, followed by a nominal group technique (NGT) focus group with the same individuals. Frequent breaks were offered, and the whole process took approximately four hours. This was run on two days with two different groups of participants, twelve in total. Nine females and three males, aged between 69 and 86 (mean age = 76), participated; all cared for a spouse of the opposite gender who was living with dementia (Alzheimer’s disease = 6, mixed dementia = 2, Parkinson’s disease dementia = 2, vascular dementia = 2). The focus groups enabled participants to discuss the supports they currently access and those they would like to see put in place. The interactional element promoted the discussion of topics that would have unlikely been raised in an individual interview, as ideas were able to develop through interaction with peers. For the NGT focus group, the six-step process was followed [28,61]. A key part of this was having participants individually rank the importance of the different types of support they identified, which resulted in an ordered list of support priorities. No previous research that had undertaken qualitative data collection over several hours such as this could be identified. We were aware of the additional burden this would put on carers, and due to known challenges in the recruitment of carers for PLWD [17,18,19], we had to implement strategies to facilitate participation. We received research funding for the conduct of this study. For a full breakdown of the NGT element of the research and the findings, please see Herron and Runacres [28]. 

Similar to previous carer research, data collection was undertaken in a conference room of a local dementia care charity [19,39]. This location had plenty of parking and public transport access to make attendance easier; it also routinely held dementia and carer support days, so it was a familiar location to some of the participants. To tackle the most significant obstacle to engagement, a lack of respite care [23,24,26], we coordinated with the dementia care charity to arrange an activity day for the individuals living with dementia in the same building. This was run by trained dementia care professionals who undertook a variety of activities with the people living with dementia so that not only were the carers engaged, but the PLWD also gained something from the day. This meant that participants were able to bring along the person they provided care for and therefore did not have to source additional care; this option was taken up by most participants.

We were aware that some carers and individuals living with dementia may feel uncomfortable in a new space with people they had not met before. Therefore, as participants were arriving with those they provided care for, we held a welcome session in which everyone had a hot drink and a snack and were able to socialise together. We built regular comfort breaks into the data collection timetable, during which the carers could leave the room and visit the PLWD. A lunch break was held in between the first and second elements of the data collection; during this, both the carers and individuals living with dementia had lunch provided for them together. It was also explained to carers that they could leave the room at any time if they needed to provide care for the PLWD. For example, in one instance, a carer needed to provide some personal care and was able to step out to do this. The provision of care for the individuals with dementia not only facilitated carer’s involvement in this research, but it also provided the carers with some time away from caring duties to engage with their peers, a form of peer support. Informal feedback from carers was positive about the social aspect of the day, and they asked for more opportunities from the charity to engage in peer support away from the person they care for. To thank participants for their time, they were provided with a £40 LovetoShop voucher at the end of the day. They were also made aware upon recruitment that additional funds were available to support participation if needed to cover costs such as specialist care or transport.

## 4. Recommendations for Qualitative Research with Carers of People Living with Dementia

In consideration of the literature critiqued and the case study presented in this article, the following recommendations are offered for researchers who intend to undertake qualitative data collection with carers of people living with dementia:

- Efforts should be made to collect data from the carer without the PLWD present.
- If alternative care is required for the PLWD, this should be built into the study design, or the costs of this for the carer should be covered.
- ‘Incidental funds’ (e.g., travel costs) should be made available so carers are not put at a financial disadvantage to participate and to ensure a representative sample.
- Sustenance (e.g., food and drinks) and remuneration for time (e.g., financial or voucher payments) should be offered to acknowledge the impact that study participation has on carers.
- Data collection should be undertaken in a neutral space, and time should be allowed for participants to acclimate prior to the commencement of data collection.

These recommendations are designed to reduce the obstacles carers of PLWD face to engaging in research [7,22]. However, we recognise challenges to their implementation. Providing respite care for individuals living with dementia built into a study design increases the legal, safety, and ethical concerns for the conduct of the research [24]. For example, researchers need to ensure that those providing the care are trained professionals, the environment in which it is provided is safe, and that appropriate governance is in place. Furthermore, due to differences in how dementia presents between individuals, types of dementia, or illness progression, providing a generic respite care service for all those cared for by participants may not be appropriate. Where this generic respite care is inappropriate, the costs of additional care arranged by the carer should be covered by the researchers. During research funding applications, costs must be outlined, research must be of good value, there are additional costs related to an inclusive research design, and ‘incidental funding’ must be available to facilitate engagement, yet the exact way in which such funds will be spent cannot be known upfront, which may be problematic for funders. Costs are further increased by the amount of time needed to design and implement inclusive research, especially for larger projects where multiple days of data collection are required. However, these costs are perhaps mitigated by the potential for a reduction in recruitment and representativeness challenges due to the removal of prohibitive factors [20,24,57,58] and the in-depth data that can be gleaned when carers are separate from those they provide care for [35].

## 5. Conclusions

Carers should be involved in the generation of the evidence necessary to develop effective supports for them [15,16]. However, the success of this relies on their ability to meaningfully engage with research, and carers face numerous obstacles to this [17,18,19]. In this article, we critically examine different approaches to qualitative data collection with carers of people living with dementia. Throughout this, we identified challenges to carer engagement with these approaches, such as additional care needs and carers not portraying an accurate representation of their experiences when the cared for individual is present. We argued that methods of data collection adopted by carers of people living with dementia should not be limited due to practical concerns as this reduces the scope and quality of the evidence base; instead, research should strive to design research projects in which carers involvement is facilitated. We presented a case study of such inclusive qualitative research and drew on this and the literature to produce recommendations for the design of inclusive research. We suggest that researchers intending to collect qualitative data with carers of a PLWD should do so without the PLWD present, build additional care into their study design or cover the costs of this for the carer, have ‘incidental funds’ available to facilitate engagement, provide sustenance and remuneration, and, finally, undertake their research in a neutral space. These recommendations are designed to facilitate the involvement of carers of PLWD in research and promote the use of more varied or multifaceted research methods to develop the current evidence base.

## Figures and Tables

**Table 1:** Summary of strengths and limitations of the methods discussed in relation to data collection with carers of people living with dementia.

Method	Strengths	Limitations
Focus groups	Collect data from multiple participants at once. Promote interaction which may identify new areas. Opportunities for peer support. Can involve a mix of participants (e.g., carers and people living with dementia)	Issues with confidentiality and anonymity. Some may feel uncomfortable sharing sensitive or personal information. Socially desirable answers may be more common. Alternative care arrangements needed to keep groups carers only.
Dyad interviews	Can collect data from carers and the person they provide care for simultaneously. The care relationship can be explored. No need for additional care arrangements.	Only relevant when the focus of the research is on both the carer and PLWD, particularly relational aspects. Some may feel uncomfortable sharing experiences in front of the person they provide care for or are cared for by. Potential for domination by one participant.
One-on-one interviews	Discussion can focus on aspects important to the individual. If undertaken in the home, there is no need for additional care arrangements. Confidentiality and anonymity concerns are reduced.	Conducting face-to-face interviews raises safety concerns. Virtual interviews exclude those with limited computer literacy. There is the potential for the PLWD to overhear and this can limit the answers provided by carers. Carer may be distracted by the needs of the PLWD. If undertaken away from the PLWD, additional care arrangements are needed.

## Data Availability

Not applicable.
